# Formation of Chlorinated Carbohydrate Degradation Products and Amino Acids during Heating of Sucralose in Model Systems and Food

**DOI:** 10.1021/acs.jafc.4c08059

**Published:** 2024-11-18

**Authors:** Michael Hellwig

**Affiliations:** † Chair of Special Food Chemistry, Technische Universität Dresden, D-01062 Dresden, Germany; ‡ Institute of Food Chemistry, Technische Universität Braunschweig, Schleinitzstraße 20, D-38106 Braunschweig, Germany

**Keywords:** sucralose, sucrose, caramelization, thermal degradation, 5-hydroxymethylfurfural, dicarbonyl compounds

## Abstract

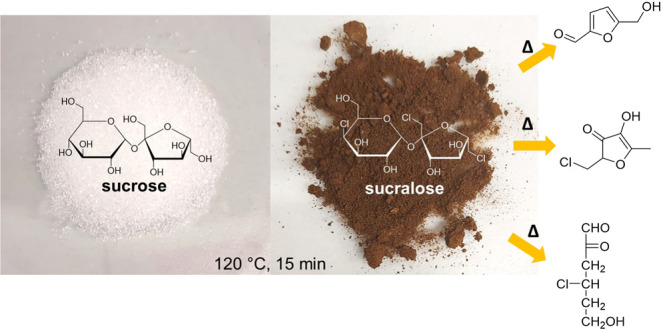

Sucralose is an artificial sweetener whose stability during the thermal treatment of food is controversially discussed. In the present work, sucralose was subjected to different kinds of heat treatment either as such, in the presence of protein, or as an ingredient of food. Compared with sucrose, sucralose showed remarkable instability and discoloration after heating at 85–90 °C for 1 h. A chlorinated furan-3-one and different chlorinated dicarbonyl compounds were identified by High-performance liquid chromatography-time-of-flight mass spectrometry (HPLC-TOF-MS) for the first time, indicating that both the 4-chlorogalactosyl residue and the 1,6-dichlorofructosyl residue give rise to novel chlorinated sugar degradation products. When sucralose was heated in the presence of protein, the formation of 3-chlorotyrosine was detected, indicating that sucralose can invoke chlorination of other biomolecules. The influence of the addition of sucralose (0.03–0.1%) to dough on pH value, color development, and HMF formation was tested in baking experiments (muffins, coconut macaroons, cookies). A significantly higher HMF concentration was observed in bakery products, including sucralose, and a chlorinated 1,2-dicarbonyl compound was detected qualitatively in baked cookies. This work shows that sucralose is not stable during baking processes at high temperatures and low moisture contents, thereby confirming recommendations from the German Institute of Risk Assessment not to use sucralose for baking.

## Introduction

Excess intake of energy from food products and its negative impact on human health have become a worldwide problem. The aim of keeping the taste of food while lowering the content of nutrients and, thereby, energy may be achieved by replacement of carbohydrates with artificial sweeteners. These compounds have a very high sweetening power and, therefore, need to be applied only in small doses in order to obtain a sweet taste. The first artificial sweetener that was discovered was saccharin in 1879.^1^ Beyond saccharin, aspartame, acesulfame-K, neotame, advantam, sucralose, neohesperidin DC, thaumatin, steviol glycosides, and alitame are generally recognized as safe (GRAS) in the USA. On the contrary, alitame is not yet permitted in the European Union. Cyclamate is permitted in the European Union but not approved for use in the USA.^2^ A recent review article concluded that artificial sweeteners can indeed help in reducing the net energy intake and, thereby, regulating body weight, whereas there is insufficient evidence for the occurrence and degree of unwanted side effects on metabolic health and the gut microbiota.^3^

**Figure 1:**
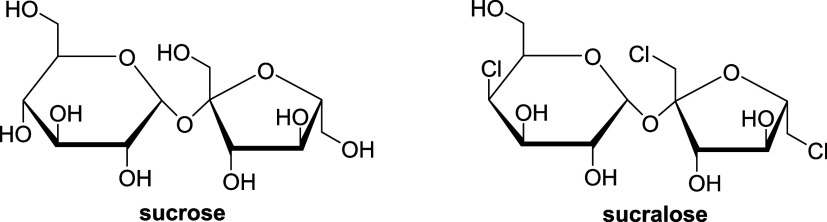
Chemical formulas of sucrose and 1,6-dichloro-1,6-dideoxy-β-d-fructosyl-4-chloro-4-deoxy-α-d-galactoside (sucralose).

Sucralose (1,6-dideoxy-1,6-dichloro-β-d-fructofuranosyl-4-deoxy-4-chloro-α-d-galactopyranoside) is a derivative of sucrose (Figure 1), with three OH groups substituted by chlorine atoms. Sucralose is produced by the chlorination of sucrose.^4^ As one of the three OH groups that is exchanged is on an optically active C atom, sucralose is a fructosyl galactoside. Its properties as a sweetener have first been described in the 1980s.^5^ It is 500–750 times as sweet as sucrose and can be applied in different energy-reduced food products.^2,6^ As for every food additive in the European Union, the toxicity of each compound has been assessed thoroughly. When consumed as a whole molecule, sucralose is not metabolized by humans, and the small amount that is absorbed is readily excreted via the kidneys.^2,5^ In the USA, the acceptable daily intake (ADI) for sucralose is 5 mg/kg body weight (bw) per day.^2^ The Joint FAO/WHO Expert Committee on Food Additives (JECFA) and the Scientific Committee on Food (SCF) derived an ADI of 15 mg/kg bw/d.^7^ The actual daily intake was estimated between 1.1 and 1.6 mg/kg of body weight (bw), with values up to 5.1 mg/kg of body weight (bw) in the 90th percentile.^2,8^ Adverse effects such as leukemia and neoplasias in mice were observed only starting from doses of ca. 250 mg/kg bw.^9^ In a randomized controlled human trial with healthy volunteers, a decrease in insulin sensitivity was observed at a dose of 0.6 mg/kg bw after a 14-day intervention.^10^

The effects of sucralose and also artificial sweeteners, in general, on the intestinal microbiota are discussed controversially. Several studies showed an adverse influence on the composition of the intestinal microbiota and inhibitory effects on quorum sensing within the microbiota.^11−13^ On the contrary, a recent placebo-controlled double-blind study with 17 healthy volunteers receiving doses of sucralose that may be reached by dietary intake (136 mg/d for 14 days, ca. 2 mg/kg bw) showed no significant changes in the composition of the gut microbiota or production of short-chain fatty acids.^14^

The commercial availability to consumers of sucralose as a sweetener may bring about an unintended use of the compound. Using sucralose as a sweetener for the purpose of omitting sucrose and other sugars in calorie-reduced baking is sometimes recommended, and sometimes it is advised against in the scientific literature.^15,16^ It is known that sucralose may liberate HCl and lead to discoloration when stored at higher temperatures.^2^ The formation of levoglucosenone during heating is due to the loss of HCl and H_2_O.^17^ The formation of a chlorinated furan compound and a chlorinated tetrahydropyran was derived from low-resolution mass-spectrometric analysis during differential scanning calorimetry experiments.^18^ The formation of polychlorinated aromatic hydrocarbons and even dioxins was suggested.^18,19^ Chloropropanols may form during the thermal degradation of sucralose in the presence of glycerol.^17^ Other authors, however, state that sucralose is safe for use also under baking conditions.^15^ This is also propagated in different online sources that address consumers (Table S1). The German Federal Institute for Risk Assessment (Bundesinstitut für Risikobewertung, BfR) concluded in 2019 that there is not enough data for a comprehensive assessment of the potential health risks of sucralose and explicitly mentions baking. BfR recommended not to use sucralose for baking, roasting, and frying.^16^

It is well-known that sucrose undergoes degradation reactions when heated in a dry state at baking temperatures, leading, among other things, to the formation of carbohydrate degradation products such as 5-hydroxymethylfurfural (HMF) and dicarbonyl compounds in a process called caramelization.^20−23^ The present work is based on the scientific question of whether chlorinated furans or dicarbonyl compounds are formed during the caramelization of sucralose. Hence, reactions of sucralose during heating and baking were investigated in comparison to those of sucrose. Degradation of both substances was followed by UV spectroscopy, TLC as well as direct High-Performance Liquid Chromatography with Ultraviolet detection (HPLC-UV) after derivatization with *o*-phenylenediamine. A baking experiment was performed to evaluate the consequences of the inclusion of sucralose in recipes of different bakery products since data on a possible formation of chlorinated compounds from sucralose under “real” baking conditions are needed.^24^

## Materials and Methods

### Chemicals and Materials

The following substances were purchased from commercial suppliers: Sucralose (powder; TCI, Eschborn, Germany); *o*-phenylenediamine (OPD) (Alfa Aesar, Karlsruhe, Germany); dialysis tubing (molecular weight cutoff (MWCO), 12 kDa), Pronase E from *S. griseus* (4000 PU/mg), dansyl chloride, TRIS, and 5-hydroxymethylfurfural (Sigma-Aldrich, Steinheim, Germany); 1-butanol, zinc sulfate heptahydrate, magnesium nitrate, sodium dihydrogen phosphate monohydrate, and sodium carbonate (Grüssing, Filsum, Germany); potassium hexacyanoferrate(II) (Riedel-de-Haën, Seelze, Germany); HPLC gradient grade methanol and acetonitrile and sulfuric acid (VWR, Darmstadt, Germany); glacial acetic acid, l-lysine, and l-valine (Roth, Karlsruhe, Germany); and water and methanol for liquid chromatography coupled to tandem mass spectrometry (LC-MS/MS) (Honeywell Specialty Chemicals, Seelze, Germany). Casein was prepared from raw cow’s milk as published previously.^25^ Double-distilled water was produced in-house (Destamat Bi 18E; QCS GmbH, Maintal, Germany). Ingredients for baking experiments were purchased from local supermarkets.

### Stability of Sucralose and Sucrose in the Dry State

Sucralose or sucrose (100 mg) was weighed into glass tubes, closed, and incubated at different temperatures (80, 90, 100, 110, 120 °C) in a preheated sand bath in a drying chamber for 1 h. After the mixture was cooled, 10 mL of water was added to each tube. The tubes were shaken and then centrifuged (8960×*g*, RT, 10 min). The complete supernatant was carefully removed, and the mixture was transferred to a new tube. The residue was dried at 50 °C in a drying chamber and weighed after cooling. The supernatants were analyzed for their pH values, UV absorbances at 280 and 430 nm, and HMF concentration. They were also subjected to derivatization with an OPD for the analysis of dicarbonyl compounds. All incubations were performed in triplicate.

### Sucralose-Protein Reactions

Mixtures of casein (100 mg) and sucralose (40 mg) or sucrose (40 mg) dissolved in 2 mL of 0.1 M sodium phosphate buffer (pH 5.5) were lyophilized and stored over a saturated solution of magnesium nitrate (*a*_W_ = 0.52)^26^ for 5 days. Samples were then either heated at 80, 120, 140, or 160 °C for 1 h. Then, samples were suspended in water and dialyzed (MWCO, 12 kDa) against distilled water for 2 days with regular changes of water. Then, the samples were lyophilized. All of the incubations were performed in duplicate.

### Baking Experiments

*Muffin* doughs (*n* = 5) were prepared from 150 g of wheat flour, 62.5 g of margarine, 1 egg (50 g), 8.5 g of tartar-based baking powder, and 60 mL of milk. Sucralose (100 mg) was added, and the dough was kneaded with a mixer. The dough was then divided into 4 portions that were placed in the cavities of a muffin pan. The muffins were baked for 20 min at 165 °C in a preheated drying chamber. *Cookie* doughs were prepared from 150 g of wheat flour, 90 g of margarine, and 25 g of egg. Sucralose (100 mg) was added, and the dough was kneaded with a mixer. After cooling for 1 h at 4 °C, the dough was rolled out (ca. 5 mm), and small cookies were cut by means of a cookie cutter (3–4 cm diameter). One part of the cookies was baked at 180 °C, the other at 220 °C for 12 min. *Coconut macaroons* were prepared from two stiffly beaten egg whites. Sucralose (150 mg) was added during whisking. Shredded coconut (100 g) was folded in the dough. The dough was divided into portions and baked at 150 °C for 15 min in a preheated drying chamber. All doughs were also prepared under the omission of sucralose for comparison.

All bakery products were ground (10 s, 20,000 U) in a laboratory mill (Tube Mill, IKA, Staufen, Germany) the following day and either directly processed or deep-frozen (−18 °C).

### Determination of the pH Value

The pH was measured with a pH meter pH 1100L (VWR, Darmstadt, Germany). Ground bakery products (10 g) were extracted with water in a volumetric flask (100 mL) and filtered. The filtrate was used for pH measurement and UV spectroscopy.

### UV Spectroscopy

This was performed with a Specord 50 plus spectrometer (Analytik Jena, Jena, Germany). The filtrate resulting from the extraction was membrane-filtered (0.45 μm) and directly analyzed. Specific coefficients of extinction (K_280_ and K_430_) were determined for 1% solutions (w/v) obtained by dissolving the residues in water after heating. If necessary, the solutions were diluted appropriately. Quartz cuvettes with the path lengths of 1 cm were used.

### Thin-Layer Chromatography (TLC)

Quantitative TLC was performed on HPTLC silica gel 60 F254 glass plates of 0.1 mm layer thickness (Merck, Darmstadt, Germany) using the solvent mixture 1-butanol/glacial acetic acid/water (8/3/3, v/v/v). Solutions were applied directly in the linear form (1–2 μL). Detection was performed by spraying the dried plate with a solution of 10% sulfuric acid in ethanol. Plates were heated to 120 °C in a drying chamber for 10 min. Calibration was performed by applying sucralose standards (*c* = 1–5 mg/L) on the same plate. Images obtained by a digital camera were evaluated densitometrically with the software ImageJ.

### Analysis of HMF

This analysis was performed with a Hitachi Elite LaChrom high-pressure liquid chromatography device with UV-detection (HPLC-UV) consisting of a pump (L-2130), an autosampler (L-2200), a column thermostat (L-2300), and a diode array detector (L-2455). A stainless steel column (250 mm × 4.6 mm, 5 μm, 100 Å) filled with Nucleodur RP-18 material (Macherey-Nagel, Düren, Germany) was used at room temperature, and 10 μL of the samples was injected. A solution of 5% acetonitrile in water served as the isocratic eluent.^27,28^ The absorbance was read at 280 nm, and UV spectra were recorded between 220 and 400 nm during the runs. Samples from heating experiments were analyzed directly after filtration (0.2 μm, regenerated cellulose). Samples from baking experiments were first clarified. To ca. 10 g of ground bakery products were added 5 mL of saturated borax solution and 25 mL of water. The samples were homogenized with an Ultra Turrax for 30–60 s, and then the mixer was rinsed with a further 25 mL of water. Then, 2 mL of Carrez I solution (15% (w/v) potassium hexacyanoferrate(II) in water) and 2 mL of Carrez II solution (30% (w/v) zinc sulfate heptahydrate in water) were added with mixing. The mixture was filled to 100 mL in a volumetric flask. After being allowed to stand at room temperature for 30 min, the mixture was filtered through a folded filter. The filtrate was membrane-filtered (0.2 μm, regenerated cellulose) and transferred to HPLC vials.

### Analysis of 1,2-Dicarbonyl Compounds

The analysis was performed with the same HPLC apparatus as that described above. As published previously,^27,28^ a stainless steel column (250 mm × 4.6 mm, 5 μm) filled with Prontosil-60 phenyl material (Knauer, Berlin, Germany) with an integrated guard column (5 mm × 4 mm) of the same material (Knauer) was used for separations at room temperature. Eluent A was 0.075% acetic acid in water, and eluent B was a mixture of eluent A and methanol (20/80, v/v). A gradient was applied (0 min, 10% B; 25 min, 50% B; 30 min, 50% B; 34 min, 70% B; 49 min, 70% B; 53 min, 10% B; 60 min, 10% B) at a flow rate of 0.7 mL/min, and the injection volume was 20 μL. The absorbance was recorded at 280, 312, and 334 nm, and UV spectra were recorded between 220 and 400 nm. For derivatization, 500 μL of samples was mixed with 150 μL of 0.5 M sodium phosphate buffer (pH 6.5) and 150 μL of a 0.2% (w/v) solution of *o*-phenylenediamine. The mixtures were stored in the dark overnight and centrifuged (8960×*g*, RT, 10 min), and the supernatants were transferred to HPLC vials.

Cookie samples were worked up as published previously.^29^ Water (3 mL) was added to 500 mg of ground samples. The samples were shaken, and then 3 mL of MeOH was added. After cooling (4 °C, 1 h), the samples were centrifuged (8960×*g*, RT, 10 min), and 500 μL of the supernatant was derivatized with OPD.

### Analysis of Amino Acids

Proteins incubated in the presence of sucralose were first hydrolyzed by enzymatic hydrolysis. TRIS buffer (0.1 M, pH 8.5, 350 μL) was added to ca. 3 mg of protein sample together with 50 μL of Pronase E solution (20 U/mL) and 20 μL of methanol. The mixture was kept at 50 °C in a drying chamber for 24 h. The samples were frozen until analysis. A blank value containing only the solutions but no protein samples was prepared as well. Derivatization to the dansyl amino acids was performed as published previously.^30^ To 100 μL of the hydrolyzates, 150 μL of 0.1 M Na_2_CO_3_ solution and 200 μL of 0.5% (w/v) dansyl chloride solution in acetone were added. After mixing, the samples were incubated at 40 °C in a water bath for 1 h. After short centrifugation, 10 μL of 3 M HCl was added. After mixing and short centrifugation, 540 μL of 0.1% aqueous formic acid was added. The samples were filtered (0.2 μm) and subjected to HPLC analysis. For calibration, solutions of lysine and valine were subjected to the derivatization procedure in concentrations between 1 and 4 mM. The analysis was performed with the same HPLC device as described above. A stainless steel column (250 mm × 4.6 mm, 5 μm) filled with Eurospher-100 C18 material (Knauer, Berlin, Germany) with an integrated guard column (5 mm × 4 mm) of the same material (Knauer) was used for separations at 40 °C. Eluent A was 0.1% formic acid in water, and eluent B was a mixture of formic acid, water, and acetonitrile (0.1/10/90, v/v/v). A gradient was applied (0 min, 20% B; 45 min, 67% B; 50 min, 20% B; 55 min, 20% B) at a flow rate of 1.0 mL/min, and the injection volume was 20 μL. The absorbance was read at 254 nm, and UV spectra were recorded between 220 and 400 nm.

### Ultrahigh-Performance Liquid Chromatography with Ultraviolet and Time-of-Flight Mass-Spectrometric Detection (UHPLC-UV-TOF-MS)

Samples obtained after incubation or, optionally, OPD derivatization were filtered (0.2 μm, regenerated cellulose), and 1 μL of samples was injected into a UHPLC system (Infinity 1290, Agilent Technologies, Waldbronn, Germany). Solvent A was 0.2% acetic acid in MS-grade water; solvent B was methanol. An RP column (Eurospher II, 100-3, 2 mm × 50 mm; Knauer, Berlin, Germany) was used at a column temperature of 25 °C. A gradient was formed (0 min, 5% B; 25 min, 90% B; 30 min, 90% B; 31 min, 5% B; 37 min, 5% B) at a flow rate of 0.2 mL/min. The absorbance was recorded at the wavelengths 280 and 312 nm. Hydrolyzed protein samples were separated on the same column using the same eluents and parameters but a different gradient (0 min, 5% B; 17 min, 50% B; 19 min, 70% B; 23 min, 5% B; 30 min, 5% B). The HPLC system was connected to the mass spectrometer TIMS-TOF (Bruker Daltonics, Bremen, Germany), working in the Scan mode (*m*/*z* 20-1300; positive mode, scan time, 500 ms; dry gas flow, 10 L nitrogen/min; dry temperature, 220 °C; nebulizer pressure, 2.2 bar; capillary voltage, 4500 V).

### High-Performance Liquid Chromatography with Triple Quadrupole Mass-Spectrometric Detection (HPLC-MS/MS)

Samples used for high-resolution mass spectrometry, either underivatized or after OPD derivatization, were also injected into an HPLC system (1260 Infinity II) coupled to the Ultivo mass spectrometer (all from Agilent). The same solvents and gradient as for HR-MS were used, but a different column (Zorbax Eclipse Plus C18, 2.1 mm × 100 mm, 3.5 μm, Agilent). The mass spectrometer worked in positive MRM mode (dry gas flow, 13 L of nitrogen/min; gas temperature, 300 °C; nebulizer pressure, 35 psi; capillary voltage, 4000 V). The fragmentor voltage was set at 100 V. The following transitions were recorded (collision energy in brackets): 163 → 145 (5 eV), 163 → 77 (10 eV), 163 → 43 (10 eV), 235 → 199 (20 eV), 235 → 193 (20 eV), and 235 → 171 (5 eV). Dwell time was 150 ms.

## Results and Discussion

### Stability of Sucralose in the Dry State

The addition of sucralose (E 955) to products with reduced energy content or without added sugar is permitted in concentrations up to 1000 mg/kg in the European Union (except chewing gum, 3000 mg/kg), including also products that undergo heat treatment such as jams, canned fruits and vegetables, and some bakery products.^31^ The German Federal Institute for Risk Assessment (Bundesinstitut für Risikobewertung, BfR) advises against heating the compound.^16^ However, it cannot be excluded that consumers use sucralose for the preparation of food under the inclusion of heating steps since there are many recipes suggesting the use of sucralose on the Internet (Table S1).

**Figure 2:**
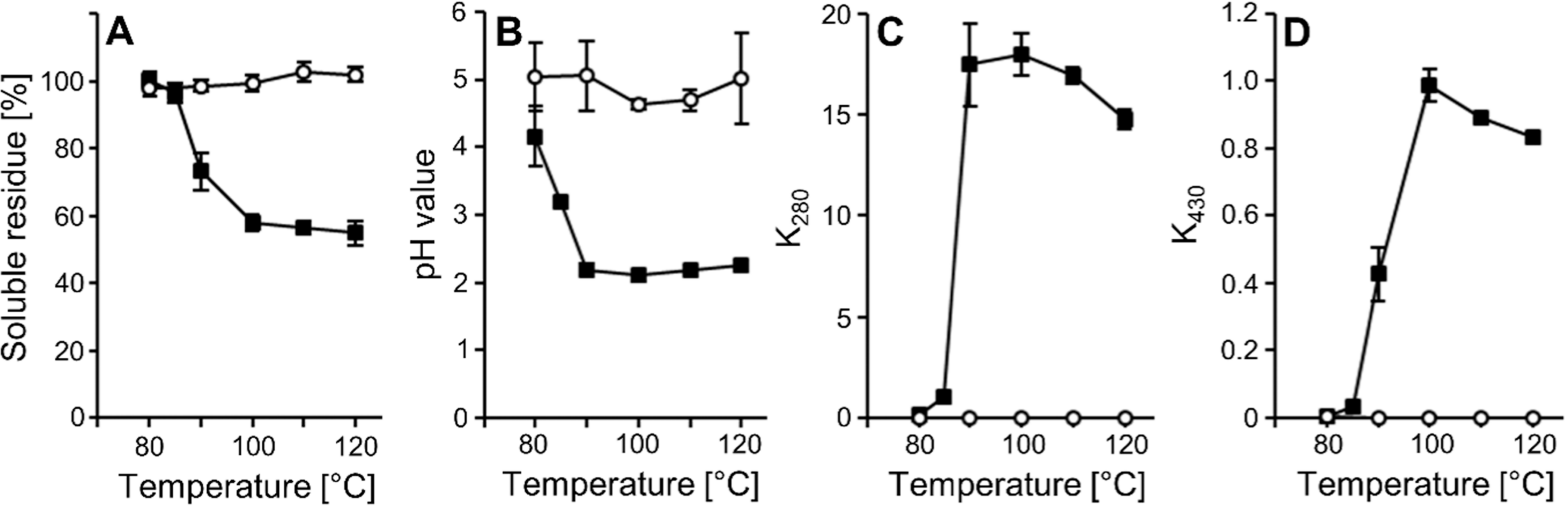
Heating of sucrose (open circles) and sucralose (closed boxes) in the dry state between 80 and 120 °C. (A) Percentage of soluble residue, (B) pH value of the dissolved residue, (C) absorption coefficient of the dissolved residue at λ = 280 nm (K_280_), and (D) absorption coefficient of the dissolved residue at λ = 430 nm (K_430_). *n* = 3–6.

In the present study, sucralose was first heated at temperatures between 80 and 120 °C in comparison to sucrose under caramelization conditions, i.e., in the absence of proteins. It became immediately visible that sucralose was less stable than sucrose because intense browning of the compound was seen already after heating at 90 °C for 1 h (Figure 2). Quantitative TLC has been used earlier in the literature for quantitation of sucralose in food.^32^ In the present work, quantitative TLC showed that sucralose was stable at 80 °C, 23.8 ± 7.7% was left at 85 °C and that the compound had disappeared after incubation at 90 °C for 1 h. In the literature, browning of sucralose starting at a temperature of 120 °C was observed during differential scanning calorimetry and thermogravimetric analysis;^18^ however, in the present work, sucralose was found to degrade already at 90 °C, probably because of the longer incubation time. Sucrose, on the contrary, did not show any browning until temperatures were up to 120 °C. The residue left after incubation was no longer completely soluble in water starting from an incubation temperature of 90 °C. Between 100 and 120 °C, only approximately 50% of the brown to black residue was still soluble in water (Figure 2). Browning was accompanied by a pungent smell of the residual material, which can be attributed to the formation of hydrochloric acid. That may have provoked the significant drop in pH in the extract of the residue seen already at a temperature of 85 °C. While the heated sucrose samples exhibited a pH value of about 5 after dissolution, the sucralose samples showed a pH of about 2 after heating at temperatures between 90 and 120 °C (Figure 2). Solutions of hydrochloric acid with pH 2.0 have a concentration of ca. 10 mM. As the residue after incubation was taken up in 10 mL of water, this would imply that at least 0.1 mmol HCl had been formed from 100 mg of sucralose (0.25 mmol).

Browning is preceded by an increase in the absorbance at 280 nm. Such a rise is also observed when glucose is heated in aqueous solution under reflux.^33^ On dry heating of sucralose, the rise in absorbance at 280 nm reaches its maximum at 90 °C and is followed by a rise in absorbance at 420 nm that reaches its maximum at 100 °C. No change in the absorbance at 280 or 420 nm was detectable when sucrose was heated (Figure 2). At 80 and 85 °C, no formation of HMF from sucralose had become visible. At higher temperatures, however, HMF concentration rose in parallel to the absorbance of the whole reaction mixture at 280 nm. Up to approximately 0.2% sucralose was converted to HMF. With a molar coefficient of extinction of HMF of 16,830 L/(mol × cm),^34^ it becomes apparent that only ca. 16% of the absorbance at 280 nm can be explained by the formation of HMF and that further UV-active compounds must have been formed.

### Identification of Chlorinated Degradation Products

**Figure 3:**
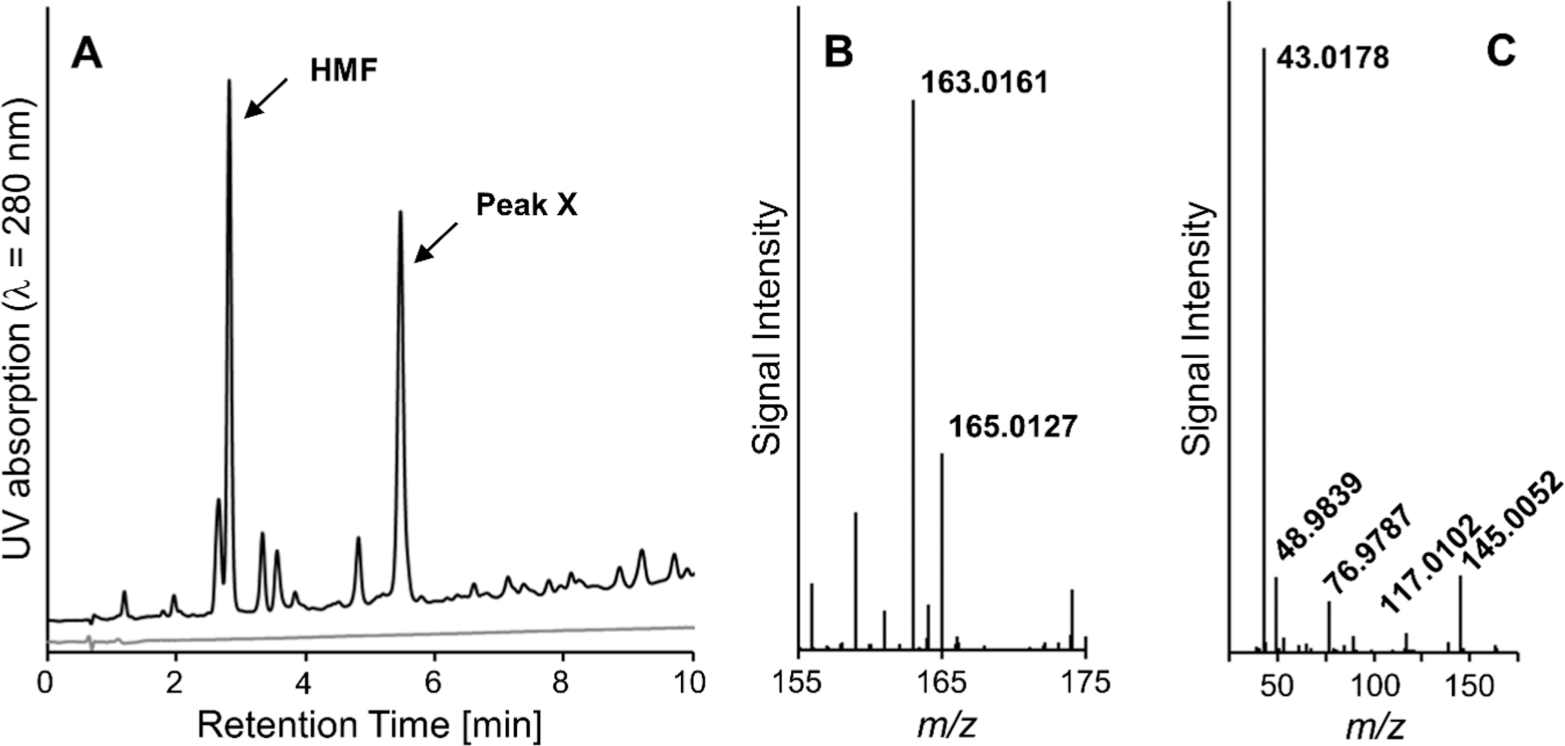
RP-HPLC with UV and mass-spectrometric detections of sucrose (gray trace) and sucralose (black trace) heated in a dry state at 120 °C for 1 h. (A) UV chromatogram recorded at λ = 280 nm. (B) The mass spectrum of Peak X. (C) The MS/MS spectrum of Peak X.

**Figure 4:**
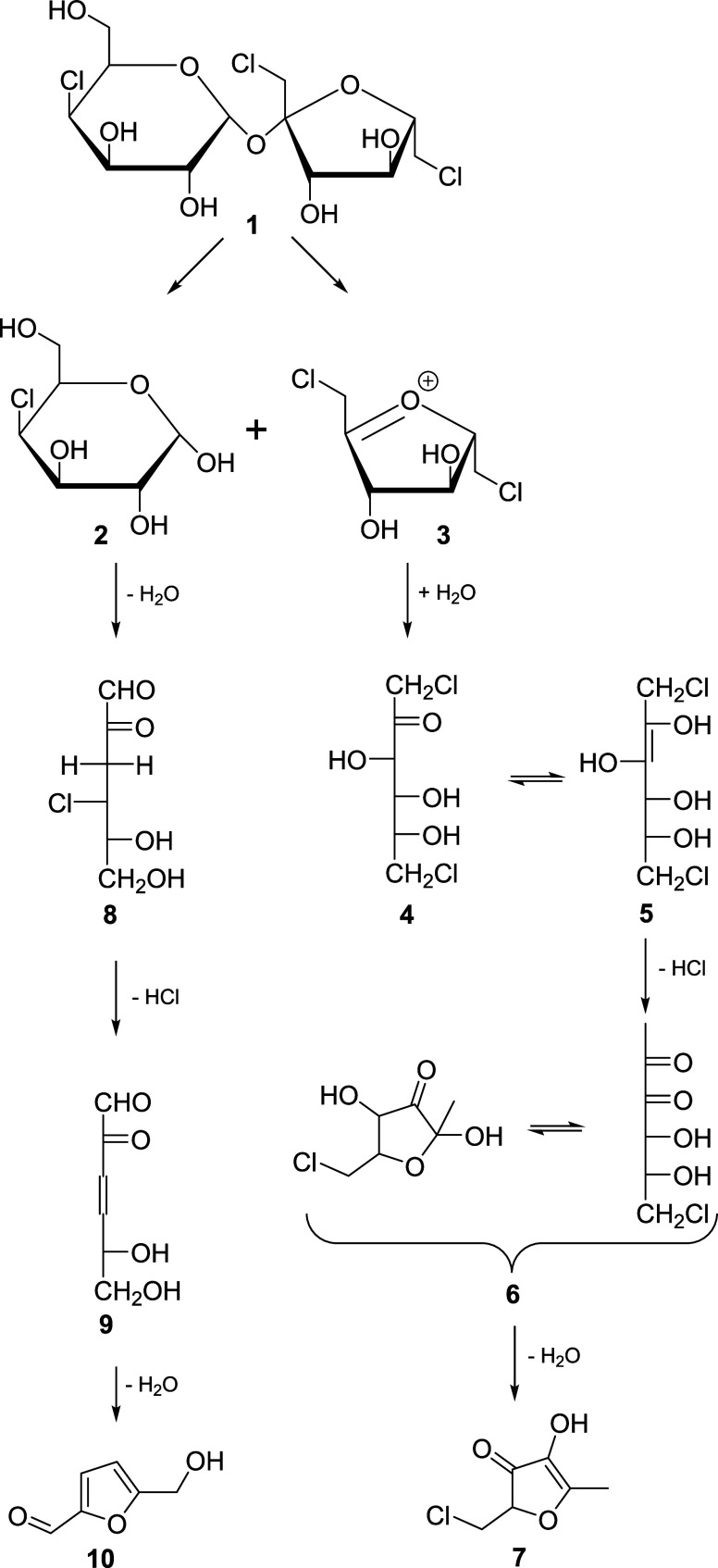
Proposed mechanism of degradation of sucralose with the formation of the products identified in the present study.

A further UV-active peak (λ = 280 nm, Peak X) eluting after HMF appeared during HPLC-TOF-MS measurements when sucralose was heated but not when sucrose was heated (Figure 3A). The peak showed a protonated pseudomolecular ion with an *m*/*z* of 163.0161. This is equivalent to the sum formula C_6_H_8_ClO_3_^+^ (*m*/*z* = 163.0157, and Δ*m*/*z* = 2.5 ppm). The presence of a monochlorinated product is verified by the characteristic ratios of the signal intensities of *m*/*z* 163.0161 as compared to *m*/*z* 165.0127 (*m*/*z*_theor._, 165.0127, Δ*m*/*z* < 0.6 ppm, Figure 3B). The ratio of 3.26 matches the natural ratio of the most abundant chlorine isotopes ^35^Cl/^37^Cl (3.13).^35^ This shows particularly well that the main second UV-active product formed during the caramelization of sucralose is a monochlorinated product. Figure 4 proposes a tentative assignment of possible structures to the signals observed here. Different groups studied the degradation of sucrose during heating and postulated the cleavage of the glycosidic bond as the main thermal degradation reaction of sucrose.^20,22,23^ Sucrose can directly decompose into HMF via the formation of a fructofuranosyl cation from its fructosyl residue. This was described as the main pathway leading to HMF from sucrose at high temperatures (250 °C).^36^ This method of formation of HMF is impossible in sucralose. Moreover, an anhydro sugar derived from fructose (2,6-anhydrofructofuranose) was postulated as the product resulting from the reaction of the C6-OH group at the cationic center at C-2.^20^ The analogous reaction of sucralose **1** would lead to the liberation of 4-deoxy-4-chlorogalactose **2** and dichlorinated fructofuranosyl cation **3**. As there is no C-6-OH group in this ion and no intramolecular attack of another hydroxyl group is possible owing to the ring strain of the resulting products, no anhydro sugar will be formed from compound **3**. After the addition of water, 1,6-dideoxy-1,6-dichlorofructose **4** would be generated, which can form a 2,3-enediol **5**. Analogously to the formation of 1-deoxygluco-2,3-diulose (1-DG) from 2,3-enolized fructose, compound **5** may lose HCl to form 1,6-dideoxy-6-chlorogluco-2,3-diulose **6**. Cyclization and loss of a molecule of water would lead to the formation of chlorinated furane-3-one **7**. The analogous furanone with an OH group instead of chlorine has been described as a reaction product during the degradation of 1-DG.^37,38^

Evidence for the postulated structure comes from the comparison of the mass spectrum of the latter hydroxy compound^37^ with the one recorded in the present study. The main fragmentation reactions of the hydroxy compound were the loss of water (−18 Da), the loss of a fragment of 46 Da, and the loss of a fragment of 60 Da.^37^ A fragment spectrum was recorded for *m*/*z* 163.0161 (Figure 3C). The fragment with the *m*/*z* 145.0052 can be explained by loss of water from the parent molecule (C_6_H_6_ClO_2_^+^; *m*/*z*_theor._, 145.0051; Δ*m*/*z* = 0.7 ppm) and the fragment with the *m*/*z* 117.0102 by loss of CO from the previous fragment (C_5_H_6_ClO^+^; *m*/*z*_theor._, 117.0102; Δ*m*/*z* < 0.9 ppm), summing up to 46.0059 Da, equivalent to the loss of 46 Da observed in the literature.^37^ The most intense mass peak with *m*/*z* 43.0178 would represent an ion resulting from the cleavage of the compound between C2 and C3 (C_2_H_3_O^+^, *m*/*z*_theor._, 43.0178; Δ*m*/*z* < 2.3 ppm). The peak with *m*/*z* 48.9839 would result from a chloromethyl ion (CH_2_Cl^+^, *m*/*z*_theor._, 48.9840; Δ*m*/*z* = 2.0 ppm) and the peak with *m*/*z* 76.9787 from the cleavage of the compound between C4 and C5 (C_2_H_2_ClO^+^, *m*/*z*_theor._, 76.9789; Δ*m*/*z* = 2.6 ppm). In the above-mentioned literature on the hydroxy analogue,^37^ a neutral loss of 60 Da was observed. The ion with *m*/*z* = 76.9787 represents the protonated chlorine analogue of that fragment. On the other hand, 4-deoxy-4-chlorogalactose **2** may dehydrate in a common pathway,^39,40^ first to 3,4-dideoxy-4-chlorogalactosone **8** and then lose HCl to form 3,4-dideoxyglucosone-3-ene **9**, ultimately resulting in HMF **10** after further dehydration. The same products might be generated when sucralose degrades via the pathway of Rahn and Yaylayan, where it is cleaved to 1,6-dichlorofructofuranose and a galactopyranosyl cation.^17^ A dichlorinated compound (bischloromethylfuran, C_6_H_6_Cl_2_O) was detected in the headspace of heated sucralose solutions.^18^ This compound, however, was not found in the present investigation.

Moreover, the formation of individual dicarbonyl compounds was investigated after the reaction with *o*-phenylenediamine (OPD). Compared to sucrose, heating of sucralose led to the formation of a multitude of UV-active peaks after derivatization with OPD. Especially chlorinated compounds were looked for, and ion chromatograms were extracted for different mono- and dichlorinated compounds. Dicarbonyl compounds can be formed by the dehydration of reducing sugars and theoretically also from compounds **2** and **4** (Figure 4). The quinoxaline of a monochlorinated dicarbonyl compound generated from a monochlorinated deoxyhexose would have the sum formula C_12_H_13_ClN_2_O_2_ (*M* = 252.0666 Da) with an *m*/*z* of the protonated monoisotopic molecular ion of 253.0738. Such a compound was found to elute after 10.2 min but only with a small abundance in the UV chromatogram recorded at λ = 312 nm, the specific wavelength for most dicarbonyl-derived quinoxalines. EIC and mass spectra are added in the Supporting Information (Figure S1). A compound bearing two chlorine atoms (C_12_H_12_Cl_2_N_2_O) was not found.

As dehydration is a common reaction of dicarbonyl compounds,^40^ ion chromatograms of singly dehydrated chlorinated species were also generated. After dehydration, a monochlorinated compound would have a sum formula of C_12_H_11_ClN_2_O with *m*/*z* of the protonated monoisotopic molecular ion of 235.0633. For comparison, the quinoxaline of 3-deoxyglucosone with its sum formula of C_12_H_14_N_2_O_3_ would show a signal with an *m*/*z* of 235.1077, which is strongly different from that of the dehydrated monochlorinated compound (Δ*m*/*z* = 189 ppm). The quinoxaline of 3-DG was not found in the reaction mixture.

**Figure 5:**
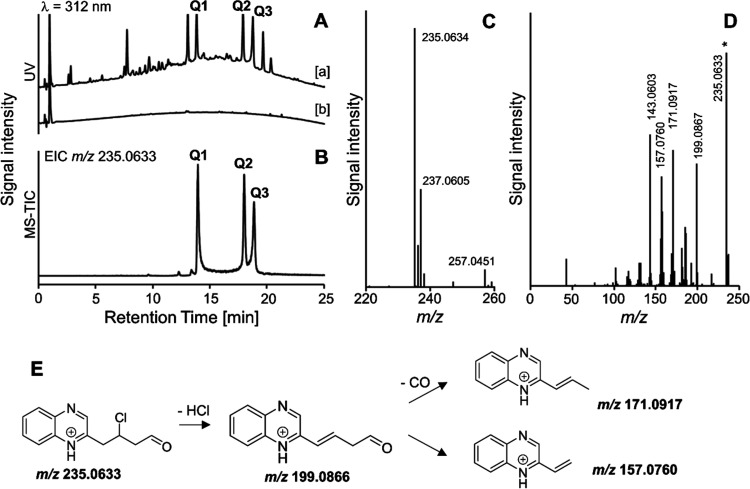
RP-HPLC with UV and mass-spectrometric detections of sucrose and sucralose heated in a dry state at 120 °C for 1 h and then dissolved in water. (A) UV chromatogram of a solution of heated sucralose (a) or sucrose (b) derivatized with *o*-phenylenediamine and (B) extracted ion chromatogram (*m*/*z* 235.0633) of a solution of heated sucralose. (C) The mass spectrum of peak Q2. (D) The MS/MS spectrum of peak Q2. (E) Proposed fragmentation of the quinoxaline of 3,4,5-trideoxy-4-chloro-6-oxo-glucosone.

Three abundant UV-active peaks showed an *m*/*z* ratio of 235.0633 (Figure 5) in the heated sucralose mixtures after OPD derivatization but not in the heated sucrose mixtures. The peak Q2 eluting at 18.0 min will be discussed more thoroughly because the respective compound was later detected in a food sample. This peak showed the characteristic ratio of the monoisotopic molecular ions of 2.7:1, as would be expected from a monochlorinated compound. The first signal (*m*/*z*_meas._ = 235.0634) deviates from the theoretical value (*m*/*z*_theor._ = 235.0633) by only Δ*m*/*z* = 0.4 ppm. The same is true for the signal containing isotope ^37^Cl (*m*/*z*_theor._ = 237.0604, *m*/*z*_meas._ = 237.0605, Δ*m*/*z* = 0.4 ppm). A sodium adduct with *m*/*z* = 257.0451 (*m*/*z*_theor._ = 257.0452, Δ*m*/*z* = 0.4 ppm) was also detected. A proposed fragmentation scheme for quinoxaline eluting at 18.0 min is shown in Figure 5E. Loss of hydrogen chloride leads to the fragment with *m*/*z*_meas._ = 199.0867 (*m*/*z*_theor._ = 199.0866, Δ*m*/*z* = 0.5 ppm). Loss of carbon monoxide generates the fragment with *m*/*z*_meas._ = 171.0917 (*m*/*z*_theor._ = 171.0917, Δ*m*/*z* < 0.6 ppm). The fragment with *m*/*z* = 143.0603 corresponds to a methylquinoxaline carbocation (C_9_H_7_N_2_^+^, *m*/*z*_theor._ = 143.0604 Δ*m*/*z* = 0.7 ppm), which has been reported as a fragment of different quinoxalines.^41−43^ These fragmentations lead to the assumption that chlorine is bound to C-4 in the sugar and that the compound is a 4-deoxy-4-chlorogalactose degradation product that might be formed from intermediate **8** by dehydration instead of dehydrochlorination (Figures 4 and S2). Attempts to isolate the quinoxaline by semipreparative HPLC failed up to now.

The MS and MS/MS spectra, together with proposed fragmentations of peaks Q1 eluting at 14.0 and Q3 eluting at 18.9 min, are shown in the Supporting Information (Figure S3). In these two spectra, a characteristic ion with an *m*/*z*_meas._ of 43.0180 is formed by α-cleavage and is an oxonium ion typical for the degradation of methyl ketones (C_2_H_3_O^+^, *m*/*z*_theor._ = 43.0178, Δ*m*/*z* = 4.6 ppm).^44^ This is the main difference in the fragmentation patterns among the three quinoxalines. The loss of CO, as in Q2, should be indicative of a quinoxaline with an oxo group at the chain end. This cleavage is possible only in Q2. In Q1 and Q3, ketomethyl groups are suggested to be present instead of an aldehyde group, which may explain the loss of the ion with *m*/*z* 43.0180 (Figure S3). Possible reactions of intermediates from Figure 4 leading to the assumed quinoxalines are shown in Figure S2.

### Reactivity of Sucralose in the Presence of Protein

Products of caramelization, such as dicarbonyl compounds, can react with nucleophilic side chains of proteins in the late stage of the Maillard reaction. The possibility of amino acid degradation and chlorination of tyrosine by reactive intermediates from sucralose degradation was analyzed in the present study. Casein was mixed with sucralose in a solution. The mixtures were lyophilized and incubated at temperatures between 80 and 160 °C for 1 h in open glass vials, which should model baking processes. After dialysis to remove the low-molecular-weight compounds, the protein residue was subjected to one-step enzymatic hydrolysis with Pronase E, which was reported to be suitable for releasing tryptophan quantitatively from protein in infant formula.^45^ The amino acids lysine and valine were quantitated by RP-HPLC-UV after dansylation, whereas 3-chlorotyrosine was measured by RP-HPLC with mass-spectrometric detection as previously,^46^ with the exception that MS was performed in the positive mode.

**Figure 6:**
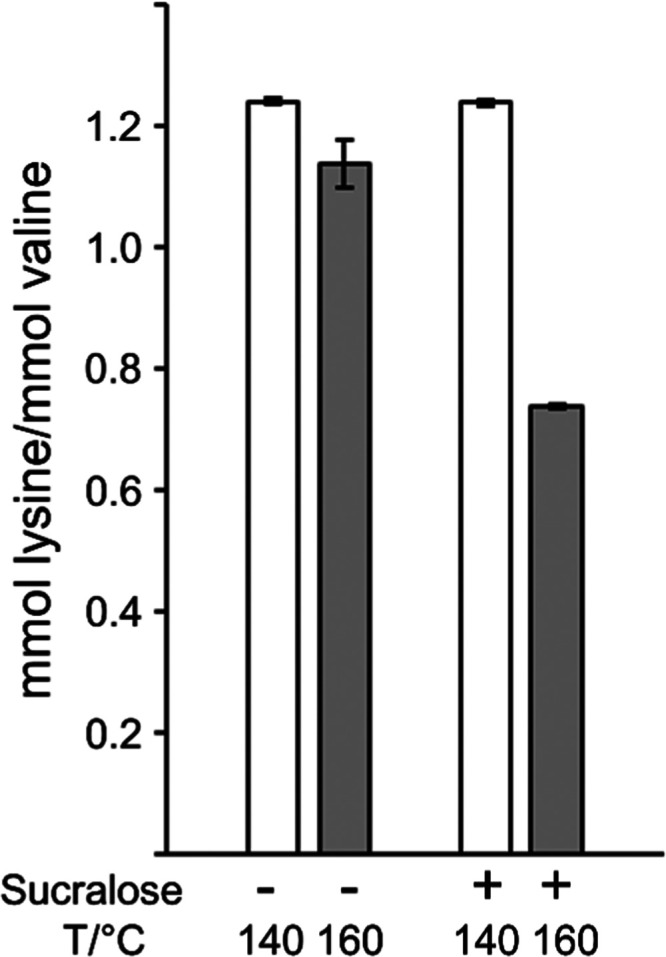
Molar ratio of lysine to valine in casein heated in the presence of sucrose or sucralose for 1 h at different temperatures.

In the presence of sucralose, proteins started to develop a brown color already at temperatures of 120 °C after heating for 1 h. Mixtures of protein and sucrose did not develop a brown color at all temperatures applied. Lysine and valine were first determined in the hydrolyzates. The absolute concentrations of lysine and valine were 71.4 g/kg casein and 60.1 g/kg casein in the sample heated in the presence of sucrose, which is very close to data from a study compiling amino acid concentrations in bovine milk obtained by different authors (lysine, 74.5 g/kg casein; valine, 65.9 g/kg casein).^47^ This points to a release of these amino acids by the enzymatic hydrolysis method of >90%. It cannot be excluded that cross-linking occurs during prolonged heating of the protein in the presence of sucralose, potentially hampering the release of individual amino acids from proteins by Pronase E. Therefore, the ratios between lysine and valine were calculated. Theoretically, the ratio between lysine and valine is between 1.1 and 1.3.^47^ The ratios measured in the incubated samples were between 1.1 and 1.2 but significantly decreased to 0.7 in the protein that had been heated at 160 °C in the presence of sucralose (Figure 6). Hence, the essential amino acid lysine can be degraded when mixtures of protein and sucralose are heated. In the same samples, more than 80% of tryptophan was degraded as well (data not shown).

**Figure 7:**
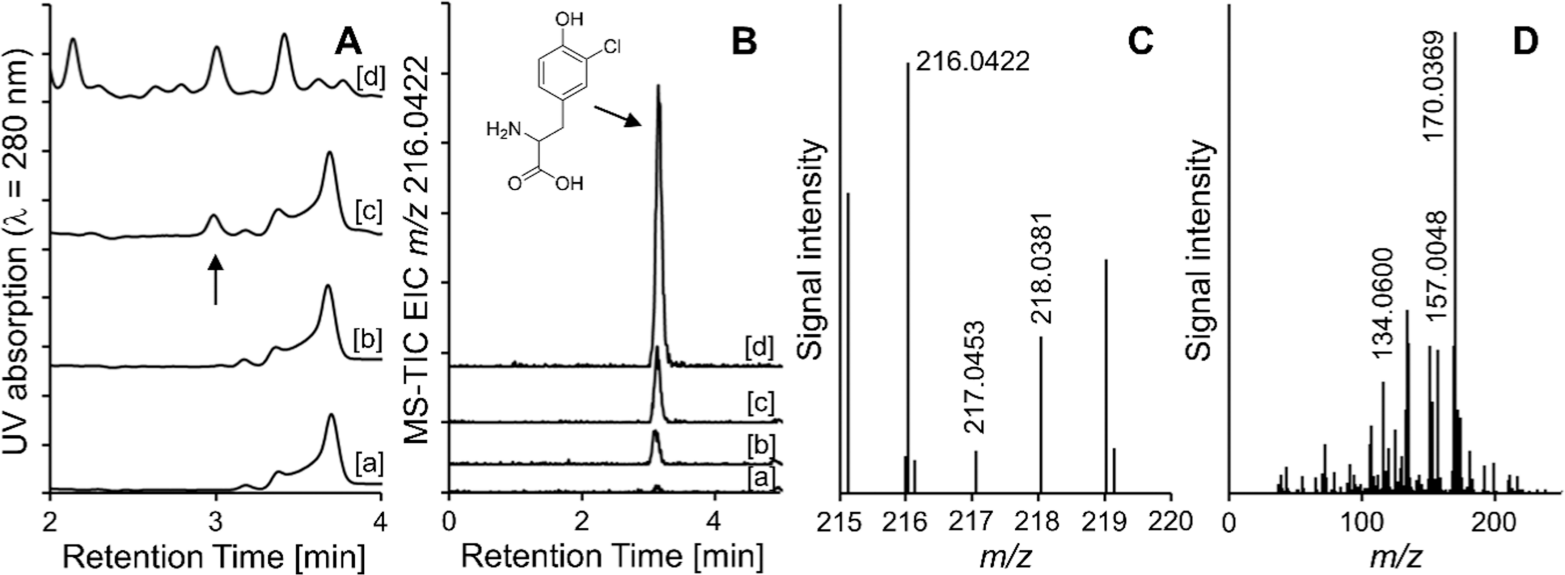
RP-HPLC with UV and mass-spectrometric detections of enzymatic hydrolysates of heated mixtures of casein and sucrose/sucralose. (A) UV chromatograms of hydrolyzates of casein samples incubated in the presence of (a) sucrose, 140 °C, (b) sucrose, 160 °C, (c) sucralose, 140 °C, and (d) sucralose, 160 °C. The arrow indicates the position of 3-chlorotyrosine. (B) Extracted ion chromatograms (*m*/*z* 216.0422) of the same hydrolyzates. (C) The mass spectrum of the peak eluting at 3.2 min in the hydrolyzed protein after coincubation with sucralose (160 °C). (D) The MS/MS spectrum of *m*/*z* 216.0422 in the same peak.

Though no significant degradation of tyrosine was observed, the formation of 3-chlorotyrosine was visible in the UV chromatograms in the samples of casein that had been heated in the presence of sucralose (Figure 7). In HPLC-TOF-MS analysis (positive mode), the compound showed a characteristic protonated monomolecular ion with an *m*/*z* of 216.0422 (*m*/*z*_theor._, 216.0422, Δ*m*/*z* < 0.5 ppm), together with a chlorine isotope signal at *m*/*z* 218.0381 (*m*/*z*_theor._, 218.0393, Δ*m*/*z* = 5.5 ppm). Mass spectra of a 3-chlorotyrosine standard are shown in the Supporting Information (Figure S4). The formation of 3-chlorotyrosine shows that the transfer of chlorine atoms to other molecules from sucralose is possible at moderately high temperatures. Chlorinated products of phenylalanine and tryptophan were searched for but not found. The incorporation of chlorine atoms into compounds heated in the presence of sucralose was already observed for glycerol, which leads to the formation of chloropropanols.^17^ However, this is the first study to show the chlorination of an amino acid in the presence of sucralose at higher temperatures.

### Stability of Sucralose during the Baking Process

**Figure 8:**
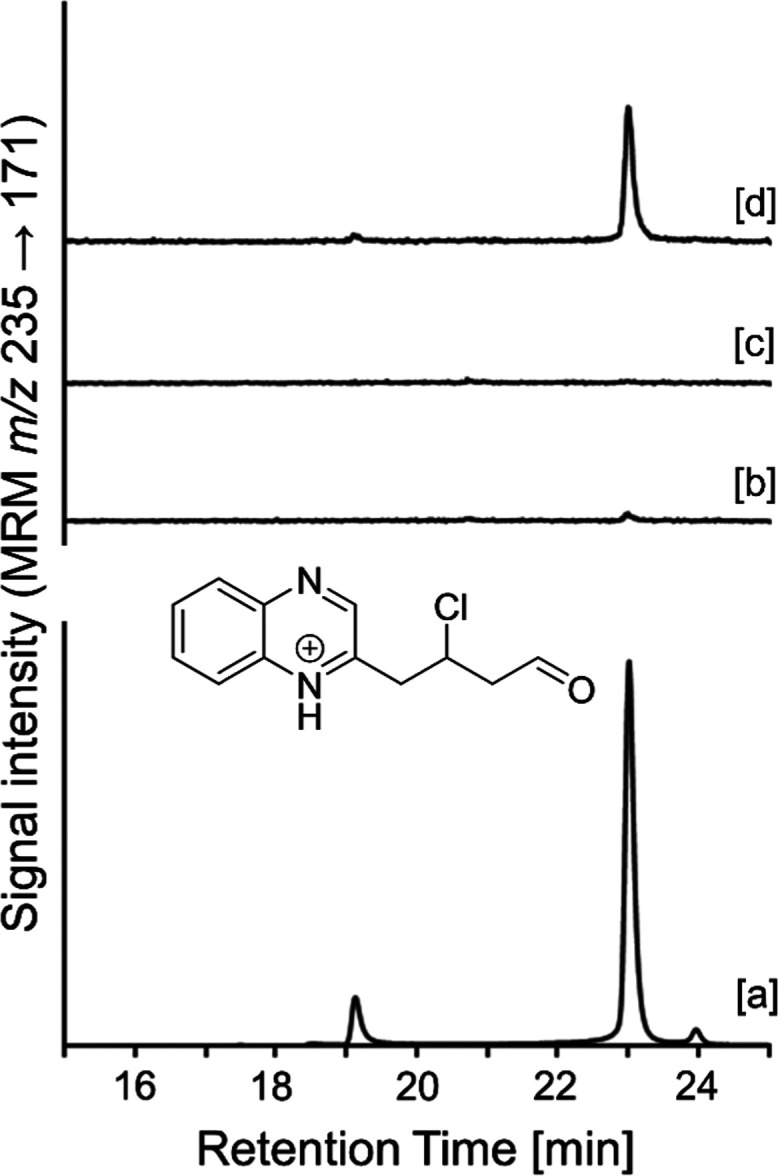
RP-HPLC-MS/MS (MRM mode) of (a) a sample of sucralose heated at 120 °C for 1 h, (b) an extract of a cookie sample baked without sucralose at 220 °C, derivatized with OPD, (c) an extract of a cookie sample baked with sucralose at 220 °C, not derivatized with OPD, and (d) an extract of a cookie sample baked with sucralose at 220 °C, derivatized with OPD. The MRM chosen was the most intense transition observed for Q2.

In different Internet sources, the use of sucralose is suggested to prepare bakery products with a reduced sugar content that retain their sweetness at the same time (Table S1). Knowledge about the formation of chlorinated compounds from sucralose in food is scarce.^24^ Therefore, baking experiments were performed with recipes that allowed the complete omission of added sugar. Two sets of dough were prepared for each recipe: one with and the other without sucralose. Any disturbances from the degradation of mono- and disaccharides that could have been observed starting from sucrose could thus be omitted. However, it was not possible to avoid side effects resulting from the degradation of starch. The model doughs were prepared without and with the addition of sucralose with 100 mg of sucralose replacing ca. 60 g of sucrose. This resulted in sucralose concentrations of 0.03–0.1% in the dough. After baking, several parameters that had already been assessed in the caramelization experiments were assessed in the ground cookie samples (Table 1). In the cookie samples containing sucralose, very little differences in pH were observed compared to the samples without sucralose. As sucralose degradation was later proven by the detection of chlorinated sugar degradation products, it can be concluded that the recognition of changes in the pH value may be inhibited by buffering effects from other ingredients, such as proteins. K_280_ was always higher in cookies baked with sucralose, but not significantly. The influence of sucralose on K_430_ was inconsistent. HMF was not observed in the muffin and coconut macaroon samples, probably due to the more alkaline pH in these doughs and the lower baking temperature as compared with the cookies. Up to 24.8 mg/kg, HMF was found in the cookies baked without sucralose (12 min at 220 °C). This HMF must have resulted from the degradation of starch. The concentration fits with concentrations reported for cookies in the literature.^48^ In the present study, it is shown for the first time that the addition of sucralose leads to a significant increase in the HMF concentrations, substantiating the results of the model experiments and the mechanism of degradation shown in Figure 4. About 2.5% of the added sucralose has been converted to HMF—similar to the caramelization experiment. This increase in the concentration of HMF cannot be due to a drop in pH because the pH was largely uninfluenced by the addition of sucralose (Table 1). The increase in HMF concentration must be traced back to the degradation of sucralose. However, only the use of isotope-labeled sucralose and the detection of isotope-labeled HMF would finally prove this hypothesis. The chlorinated furan-3-one **7** (Figure 4) was not detected in the HPLC-TOF-MS measurements. In order to circumvent the low sensitivity of HPLC-TOF-MS measurements, an HPLC-MS/MS method assessing the putative chlorinated furanone and the characteristic dicarbonyl compounds was developed. This method also did not allow the detection of chlorinated furan-3-one **7**. However, quinoxaline Q2 was detected by the characteristic transitions 235 → 199 (loss of HCl) and 235 → 171 (loss of HCl and CO, Figure 5) in cookie samples baked at 220 °C with sucralose (Figure 8). This shows for the first time that chlorinated dicarbonyl compounds can be formed during baking under the inclusion of sucralose.

**Table 1:** pH Values, Specific Extinction Coefficients, and HMF Concentrations of Different Model Bakery Products Baked with or without Sucralose^a^

		muffin		coconut macaroon		cookie		cookie	
sucralose		+	−	+	−	+	−	+	−
baking temp.	[°C]	170	170	150	150	180	180	220	220
baking time	[min]	20 min	20 min	15 min	15 min	12 min	12 min	12 min	12 min
pH		8.27 ± 0.20	8.34 ± 0.16	7.27 ± 0.01	7.22 ± 0.03*	6.88 ± 0.02	6.93 ± 0.01*	6.63 ± 0.02	6.61 ± 0.07
K_280_		1.8 ± 0.2	1.7 ± 0.1	5.4 ± 0.4	5.0 ± 0.3	0.81 ± 0.04	0.79 ± 0.01	2.3 ± 0.3	2.2 ± 0.2
K_430_		0.12 ± 0.04	0.11 ± 0.03	0.13 ± 0.02	0.11 ± 0.02	0.02 ± 0.01	0.04 ± 0.02*	0.07 ± 0.01	0.09 ± 0.03
HMF	[mg/kg]	n.d.	n.d.	n.d.	n.d.	0.84 ± 0.27	0.26 ± 0.04*	33.3 ± 4.7	24.8 ± 3.1*

a Data are presented as means ± SD (*n* = 4–9). * implies that means are statistically significantly different in cookied with and without addition of sucralose (*P* < 0.05) as determined by a *t*-test. n.d., not detectable.

To sum up, the present study shows that sucralose is unstable during comparatively mild heating, leading to the formation of chlorinated sugar degradation products and chlorination of other biomolecules. Sucralose participates in the caramelization processes. Further studies are necessary that focus on the stability of sucralose under conditions that can be expected from consumer behavior under the inclusion of well-known process contaminants such as chloropropanols.^17^ The easy availability of certain food additives, together with possible unexpected use by consumers, who may not be aware of the individual permissions for the use of additives, may represent a poorly regarded aspect in the toxicological risk assessment of food additives. During toxicological risk assessment, the stability and reactivity of additives should be considered more thoroughly in the case of unanticipated use, which has become more likely due to the easy availability of isolated additives for the consumer.

## Supporting Information Available

Internet sources propagating the use of sucralose for baking (Table S1); chromatographic and MS results for the identification of a quinoxaline of a monochlorinated deoxyosone (Figure S1); proposed pathways for the formation of chlorinated dicarbonyl compounds Q1–Q3 (Figure S2); MS and MS/MS spectra of quinoxalines Q1 and Q3 including proposed fragmentations (Figure S3); and MS and MS/MS spectra of a chlorotyrosine standard (Figure S4) (PDF)

## Abbreviations

1-DG: 1-deoxygluco-2,3-diulose
ADI: acceptable daily intake
BfR: Bundesinstitut für Risikobewertung (German Federal Institute for Risk Assessment)
bw: body weight
EIC: extracted ion chromatogram
FAO: food and agriculture organization
HMF: 5-hydroxymethylfurfural
HPLC: high-performance liquid chromatography
JECFA: Joint FAO/WHO Expert Committee on Food Additives
MRM: multiple reaction monitoring
MWCO: molecular weight cutoff
OPD: *o*-phenylenediamine
RT: room temperature
SCF: scientific committee on food
TLC: thin-layer chromatography
TOF: time-of-flight
TRIS: tris(hydroxymethyl)aminomethane
WHO: world health organization
