# Supplementary material for: Formation of Chlorinated Carbohydrate Degradation Products and Amino Acids during Heating of Sucralose in Model Systems and Food
Source: J Agric Food Chem. 2024 Nov 18;72(47):26441–50. doi: 10.1021/acs.jafc.4c08059 (PMC11613498; doi:10.1021/acs.jafc.4c08059)
Supplement: Supplementary file 1 — jf4c08059_si_001.pdf [file jf4c08059_si_001.pdf]

Formation of Chlorinated Carbohydrate Degradation Products and Amino Acids during Heating of Sucralose in Model Systems and Food

Michael Hellwig[1,2,*]

[1] Chair of Special Food Chemistry, Technische Universität Dresden, D-01062 Dresden, Germany

[2] Institute of Food Chemistry, Technische Universität Braunschweig, Schleinitzstraße 20, D-38106 Braunschweig, Germany

M. Hellwig

Technische Universität Dresden

Chair of Special Food Chemistry

Bergstraße 66

D-01062 Dresden, Germany

Tel.: +49-351-463-32006

E-mail: Michael.Hellwig@tu-dresden.de

Table S1. Internet Sources Propagating the Use of Sucralose for Baking.

URL	Date
https://super-twins.de/2022/05/15/kuenstliche-suessstoffe-sucralose-erhitzen/?cookie-state-change=1724355557444 [German]	22 august 2024
https://www.allrecipes.com/article/baking-with-sugar-and-sugar-substitutes/	22 august 2024
https://chas.org/blog/baking-and-sugar-substitutes/	22 august 2024
https://www.acefitness.org/resources/everyone/blog/6193/baking-with-sugar-substitutes-which-ones-are-good-for-baking/	22 august 2024
https://www.splenda.com/baking-tips-for-splenda-original-sweeteners/	22 august 2024
https://www.bbc.co.uk/food/sucralose	22 august 2024
https://morenutrition.de/blogs/wissenswertes/suessungsmittel-mythen [German]	22 august 2024

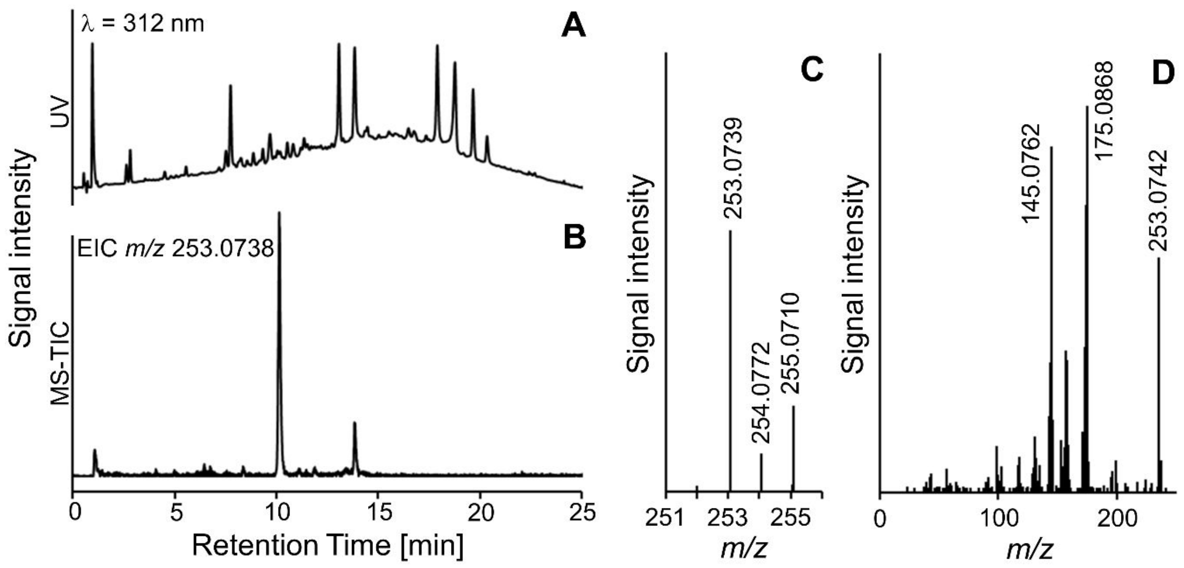

Figure S1. RP-HPLC with UV and mass-spectrometric detections of sucralose heated in dry state at 120 °C for 1 h and then dissolved in water. (A) UV chromatogram of a solution of heated sucralose derivatized with *o*-phenylenediamine and (B) Extracted ion chromatogram (*m/z* 253.0738) of the same solution. (C) Mass spectrum of the peak eluting at 10 min. (D) MS/MS spectrum of the peak eluting at 10 min.

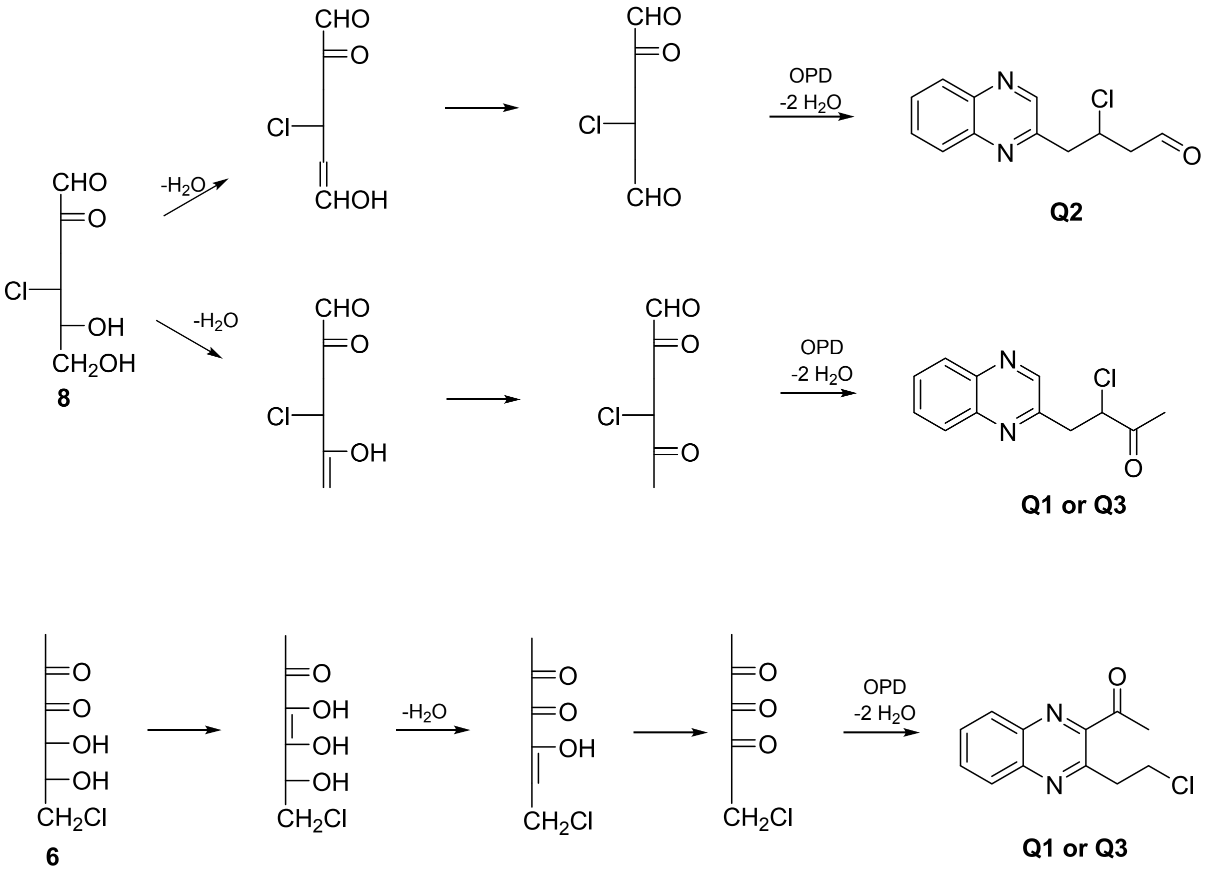

Figure S2. Proposed pathway of formation of quinoxalines of chlorinated dicarbonyl compounds from caramelization reaction of sucralose (cf. Figure 4 in the main text).

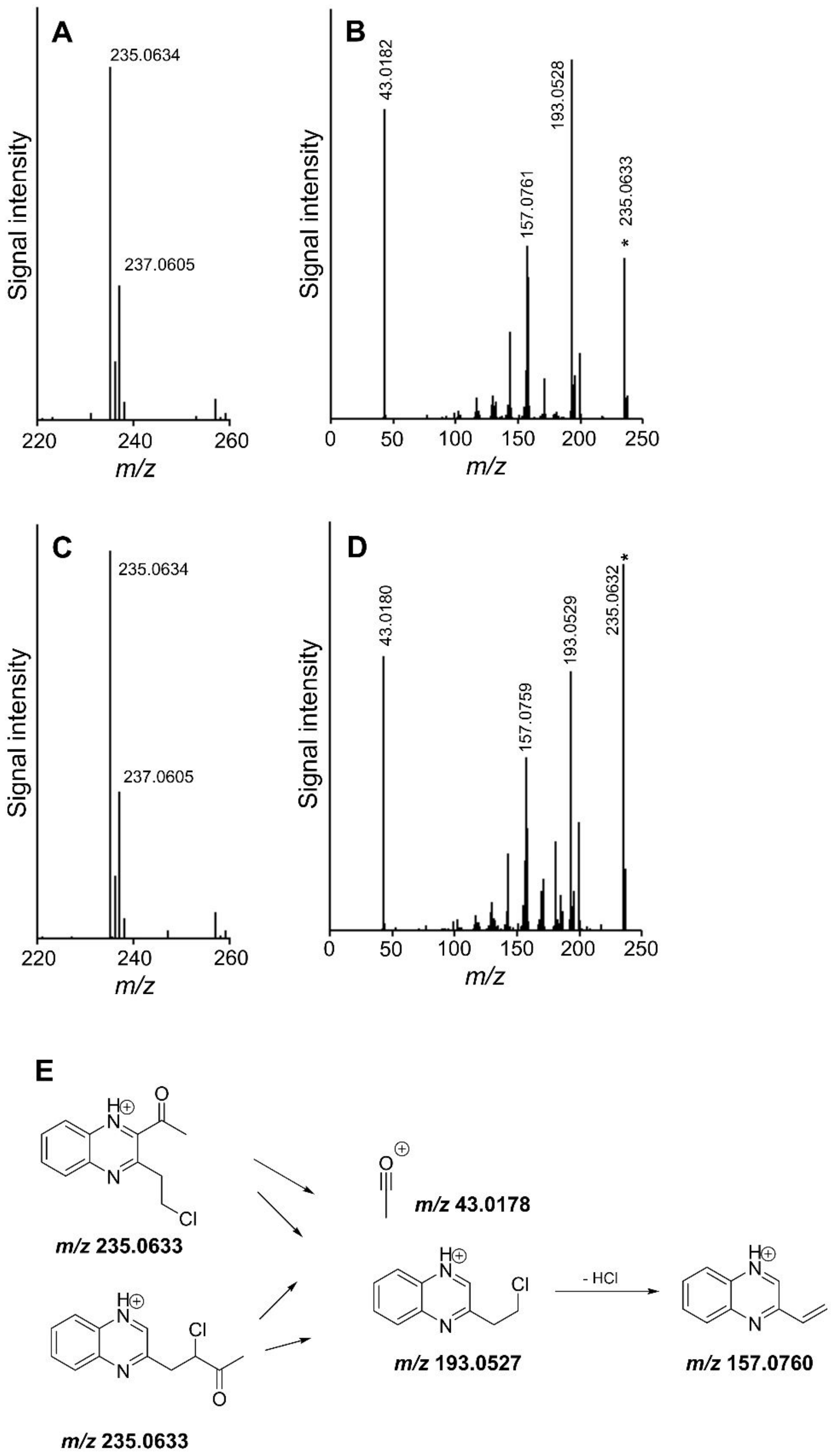

Figure S3. (A) Mass spectrum of peak Q1. (B) MS/MS spectrum of peak Q1. (C) Mass spectrum of peak Q3. (D) MS/MS spectrum of peak Q3. (E) Proposed fragmentation of the quinoxalines of 2,3,4-trioxo-6-chlorohexane and 3,4,6-trideoxy-4-chloro-5-oxo-glucosone.

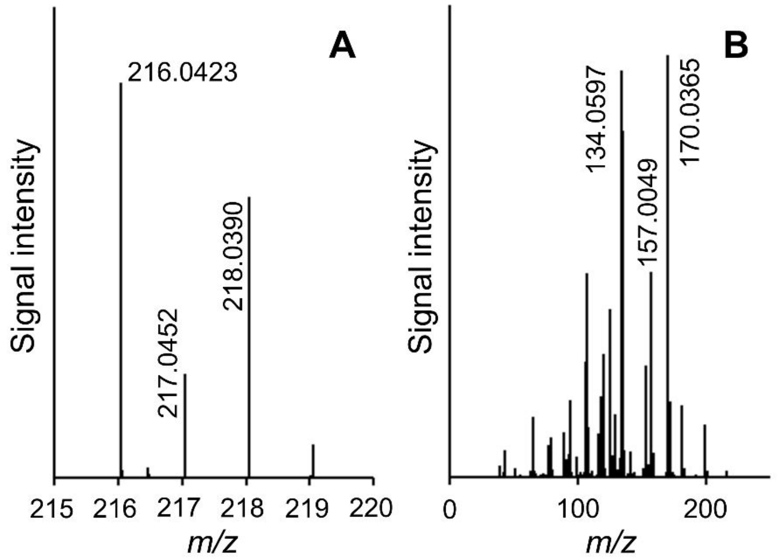

Figure S4. (A) MS and (B) MS/MS spectra of a chlorotyrosine standard measured by RP-HPLC with TOF-MS detection (positive mode).
